# Remimazolam as a Novel Anesthetic in Pediatric Procedural Sedation and Anesthesia: A Narrative Review

**DOI:** 10.7759/cureus.86354

**Published:** 2025-06-19

**Authors:** Min Zhu, Miaomiao Tang

**Affiliations:** 1 Department of Anesthesiology, West China Hospital of Sichuan University, Chengdu, CHN

**Keywords:** general anesthesia, narrative review, pediatric patients, pharmacological characteristics, procedural sedation, remimazolam

## Abstract

As an ultra-short-acting benzodiazepine, remimazolam demonstrates rapid onset, quick offset, and a short half-life. Current evidence suggests that its metabolism is not dependent on hepatic or renal function, and its effects can be reversed by flumazenil, a benzodiazepine antagonist. Ever since its approval for procedural sedation and general anesthesia, it has gained popularity due to its minimal impact on circulation and respiration and reduced incidence of adverse reactions. While the clinical application of remimazolam in adult and elderly patients has been studied and reported extensively, its safety and efficacy in pediatric patients remain unclear. Current evidence suggests its potential clinical value and application prospects in children. This review outlines the pharmacokinetics and pharmacodynamics of remimazolam and summarizes the latest research progress regarding its use in pediatric patients for procedural sedation and anesthetic induction and maintenance. We conducted a literature search using PubMed and the China National Knowledge Infrastructure (CNKI), specifically targeting clinical studies and case reports that evaluated the use of remimazolam for procedural sedation and general anesthesia in pediatric patients. The search strategy incorporated the following keyword combinations: "remimazolam and pediatric anesthesia", "remimazolam and pediatric sedation", "remimazolam and children", and "remimazolam and pediatric patients". In summary, we synthesized existing clinical studies and case reports to provide guidance and insights into the use and research of remimazolam in pediatric patients.

## Introduction and background

Every year, millions of children undergo anesthesia for surgical operations as well as diagnostic and therapeutic procedures [1,2]. However, pediatric patients are more vulnerable to conditions such as cyanosis, bradycardia, and cardiac arrest during sedation and general anesthesia due to their inherently limited cardiopulmonary functional reserves and the inhibitory effects of anesthetic drugs on circulation and respiration [3,4]. Besides, mounting studies in recent years suggest that single long exposures and multiple exposures to general anesthesia can adversely affect the neurodevelopment of children, emphasizing the potential neurotoxicity of anesthetics and sedatives [5]. Therefore, the identification and selection of anesthetic agents with minimal adverse effects is paramount to ensure pediatric patient safety.

At present, the commonly used sedative and anesthetic drugs include propofol, sevoflurane, midazolam, and dexmedetomidine (Table 1). Propofol, favored for its rapid onset of action and recovery, minimal accumulation, and low incidence of side effects such as postoperative nausea and delirium, remains the most widely utilized anesthetic agent for sedation and anesthesia purposes. However, it has significant inhibitory effects on the respiratory and cardiovascular systems, which can be exacerbated in elderly or critical patients [6]. Moreover, cases of adverse neurological effects related to propofol infusion have been reported in pediatric patients [7-9]. Sevoflurane can increase the risk of agitation or delirium in pediatric patients [10]. Besides, circulatory depression and prolonged action times restrict the application of midazolam and dexmedetomidine.

**Table 1:** Drugs for procedural sedation and general anesthesia

Drug	Onset time	Terminal half-life	Advantages	Adverse effects
Remimazolam	<5 min	37-53min	Ultrashort-acting, predictable; hemodynamically stable; minimal respiratory depression; flumazenil-reversible	Risk of re-sedation following flumazenil reversal of remimazolam; acute tolerance?
Propofol	<1 min	30-60 min	Fastest onset; rapid, clear recovery; antiemetic properties	Significant respiratory/cardiovascular depression; injection pain; lipid metabolism risk; propofol infusion syndrome
Sevoflurane	1-2 min	30-60 min	Inhalation advantage (difficult airway); rapid recovery	Malignant hyperthermia risk; dose-dependent circulatory depression emergence delirium
Midazolam	2-3 min	1.5-3 h	Excellent anterograde amnesia; fastest onset	Respiratory depression; paradoxical excitement; delayed recovery
Dexmedetomidine	5-10 min	2-3 h	Unique "arousable sedation"; no respiratory depression; reduces postoperative delirium; analgesic adjunct	Bradycardia/hypotension; slow onset; delayed recovery (high doses)

In recent years, remimazolam, a novel anesthetic, has been widely employed for procedural sedation and the induction and maintenance of general anesthesia in adults. This benzodiazepine drug has many advantages, including a short onset of action and fast recovery. Furthermore, its anesthetic efficacy is not inferior to that of propofol. Studies have demonstrated that in comparison to propofol, remimazolam reduced the incidence of injection pain, cardiovascular or respiratory inhibition, and postoperative delirium [11]. Although remimazolam has not yet received approval for pediatric use from the U.S. Federal Drug Administration (FDA), mounting research and reports have explored its safety, efficacy, and application potential in pediatric patients undergoing procedural sedation or anesthesia. This paper expounds on the pharmacological properties of remimazolam and reviews the progress in research on remimazolam use in pediatric patients undergoing procedural sedation or anesthesia. Importantly, we sought to provide a clinical reference for its application in pediatric patients and identify future research directions.

## Review

Methods

We conducted a literature search using PubMed and the China National Knowledge Infrastructure (CNKI), specifically targeting clinical studies and case reports that evaluated the use of remimazolam for procedural sedation and general anesthesia in pediatric patients. The search strategy incorporated the following keyword combinations: "remimazolam and pediatric anesthesia", "remimazolam and pediatric sedation", "remimazolam and children", and "remimazolam and pediatric patients". No time restrictions were applied. We included randomized controlled trials (RCTs), prospective and retrospective cohort studies, systematic reviews, and case reports regarding the use of remimazolam in pediatric patients (Table 2). The exclusion criteria consisted of studies published in languages other than English or Chinese.

**Table 2:** Key studies of literature review CT, computed tomography; MRI, magnetic resonance imaging; ED95, 95% effective dose; ED50, 50% effective dose; PACU, post-anesthesia care unit; MH, malignant hyperthermia; MELAS, mitochondrial encephalomyopathy, lactic acidosis, and stroke-like episodes; DMD, Duchenne muscular dystrophy; GA-1, glutaric acidemia type I; MCADD, medium-chain acyl-CoA dehydrogenase deficiency

Research design and sample size	Application	Conclusions	Reference
Prospective; N=24; age=2-6 years; intravenous remimazolam	General anesthesia with sevoflurane	Remimazolam in pediatric patients shows high clearance and rapid context-sensitive half-time	[12]
Prospective; N=60; mean age=2.8 ± 1.3 years; intravenous remimazolam, propofol	Procedural sedation; CT/MRI examinations	Compared with propofol, remimazolam offers effective sedation with a faster recovery time and exerts minimal effects on children's cardiovascular and respiratory functions	[13]
Prospective; N=23; age=5-18 months; intravenous remimazolam, intranasal dexmedetomidine	Procedural sedation; echocardiographic examination	Compared with dexmedetomidine, intravenous remimazolam exhibits significantly better sedative effects with shorter recovery time for pediatric echocardiography	[14]
Prospective; N=30; age=1-6 years; intravenous remimazolam, propofol	Procedural sedation; painless gastroscopy	Remimazolam demonstrates significantly lower adverse reaction rates than propofol in painless gastroscopy; however, its optimal dose regimen requires further investigation	[15]
Prospective; N=40; age=6-15 years; intravenous remimazolam, propofol	Procedural sedation; painless gastroscopy	Remimazolam demonstrates less adverse reaction, and combination of remimazolam and alfentanil provides a safe and effective anesthesia solution for pediatric gastroscopy procedures	[16]
Prospective; N=40-42; age=3-12 years; intravenous remimazolam, propofol	Procedural sedation; painless gastroscopy	A dose of 0.4 mg/kg remimazolam not only increases sedation success rates but also reduces the need for rescue sedation and adverse reactions	[17]
Prospective; N=50 or 56; age=7-12 years; combining remimazolam with propofol, intravenous propofol	Procedural sedation; painless gastroscopy	Combining remimazolam with propofol for pediatric gastroscopy ensures effective sedation while reducing adverse effects, stabilizing hemodynamics, and accelerating recovery	[18]
Prospective; N=30; age=6-12 years; combining remimazolam with propofol, intravenous remimazolam, propofol	Procedural sedation; fiberoptic bronchoscopy	The combination of remimazolam and low-dose propofol balances the benefits and limitations of both agents, providing more stable hemodynamics and fewer adverse reaction in pediatric fiberoptic bronchoscopy	[19]
Prospective; N=35; age=2-6 years; combining remimazolam with propofol, intravenous propofol	Procedural sedation; root canal therapy	Remimazolam-propofol combination provides better sedation than propofol alone for pediatric outpatient root canal therapy	[20]
Prospective; N=40; Age=1-6 years; intranasal remimazolam	Procedural sedation; premedication	The ED95 dose of a single intranasal administration of remimazolam for effectively relieving preoperative anxiety is 1.57 mg/kg in early childhood children and 1.09 mg/kg in preschool children	[21]
Prospective; N=23; age=1-6 years; intravenous remimazolam,	Procedural sedation; premedication	For preoperative sedation in children aged 1-6 years, the ED50 and ED95 of remimazolam are 0.051 and 0.077 mg/kg, respectively	[22]
Prospective; N=32; age=3-6 years; intranasal remimazolam	Procedural sedation; premedication	The ED50 and ED95 of intranasal remimazolam for pre-induction sedation are 0.65 and 0.78 mg/kg, respectively	[23]
Prospective; N=30-31; age=1-4 years; intravenous remimazolam, esketamine	Procedural sedation; premedication	In pediatric preoperative sedation, 0.2 mg/kg intravenous remimazolam has a slightly longer onset time than 0.5 mg/kg esketamine but offers faster recovery and more stable hemodynamics	[24]
Retrospective; N=418; mean age =4.6 years; intravenous remimazolam, esketamine	General anesthesia	Remimazolam may promote faster emergence from general anesthesia	[25]
Prospective; N=140 or 47; age=3-6 years; intravenous remimazolam, propofol	General anesthesia	Remimazolam is well tolerated and non-inferior to propofol for induction and maintenance of general anesthesia in preschool children	[26]
Prospective; N=40; age=5-8 years; intravenous remimazolam, propofol	General anesthesia; dental day surgery	Remimazolam demonstrates superior safety and recovery outcomes in pediatric dental day surgery	[27]
Prospective; N=40; age=3-7 years; intravenous remimazolam, propofol	General anesthesia; dental day surgery	Compared with propofol, remimazolam significantly reduces extubation time and PACU stay duration in pediatric dental day surgery and decreases emergence agitation during recovery	[28]
Prospective; N=40; age≥3 years; intravenous remimazolam, propofol	General anesthesia; tonsillectomy and adenoidectomy	Compared with propofol, remimazolam has a milder impact on the cardiovascular system, reduces postoperative complications, decreases emergence agitation, and provides better pain relief	[29]
Prospective; N=40; age=1-6 years; intravenous remimazolam, saline	General anesthesia; laparoscopic inguinal hernia repair	Both continuous infusion and a single bolus of remimazolam effectively reduce the incidence of emergence delirium in children	[30]
Meta-analysis; N=310; age=1-7 years; intravenous remimazolam, saline	General anesthesia; tonsillectomies and adenoidectomies, laparoscopic inguinal hernia repairs, and dental procedures	Remimazolam may reduce the incidence of emergence delirium following pediatric anesthesia without significantly prolonging emergence time	[31]
Prospective; N=30; age=2-10 years; intravenous remimazolam, propofol	General anesthesia; cardiac surgery	Remimazolam-flumazenil combination promotes early extubation, shortens hospitalization, and lowers costs in pediatric fast-track cardiac surgery without increasing adverse events	[32]
Case report; age=6 years; combining remimazolam with propofol	General anesthesia; dental rehabilitation procedure; suspected family history of MH	Remimazolam demonstrates potential safety as an adjunct to propofol for general anesthesia in pediatric patients with suspected MH susceptibility	[33]
Case report; age=4-10 years; intravenous remimazolam	General anesthesia; MELAS, DMD, GA-1, or MCADD	Remimazolam demonstrates safety and efficacy for both induction and maintenance of general anesthesia in children with MELAS, DMD, GA-1, or MCADD	[34-37]

Mechanism of action

Remimazolam is a novel short-acting benzodiazepine that shares a similar chemical structure with midazolam (i.e., incorporating a metabolizable methyl propionate side chain into the benzodiazepine master ring of midazolam). Its mechanism, similar to midazolam, is to enhance receptor activity to induce cell membrane hyperpolarization and decrease excitability via acting on γ-aminobutyric acid A (GABAA) receptor, thereby inhibiting neural electrical activities and producing sedation, hypnosis, anti-anxiety, and anterograde amnesia. Its sedative effects can be reversed by flumazenil [38]. Interestingly, similar to remifentanil’s metabolic pathway, remimazolam is mainly decomposed by tissue esterases (mainly carboxylesterase 1) into the inactive carboxylic acid metabolite CNS7054, which confers the advantages of rapid onset and offset and metabolic independence from hepatic and renal function [39,40].

Pharmacokinetics

In a study involving single increasing doses of remimazolam administered to healthy subjects, the pharmacokinetic model showed that remimazolam exhibited a high systemic clearance (overall mean, 70.3±13.9 L/h, mean±SD), moderate volume of distribution (34.8±9.4 L), and a short terminal half-life (0.75±0.15 h). Its metabolite, CNS7054, demonstrated slower clearance (4.22±0.65 L/h), smaller volume of distribution (17.5±3.8 L), and longer terminal half-life (2.89±9 h) in comparison with the parent compound [39]. In severe hepatic insufficiency patients, the clearance of remimazolam was 38.1% lower, while in patients with moderate hepatic impairment and renal impairment, plasma clearance of remimazolam was comparable to healthy subjects. The pharmacokinetics of remimazolam were linear within the studied dose range and were reportedly not affected by chronic renal disease, age, race, or weight. Besides, population pharmacokinetic studies of remimazolam in children have been investigated. Gao et al. [12] recruited 24 children aged 3-6 years undergoing general anesthesia with sevoflurane, where remimazolam was administered via intravenous infusion at 5 mg/kg/h for 5 min, followed by 1.5 mg/kg/h for 55 min during surgery. They used a three-compartment model and a two-compartment model to describe the pharmacokinetics of remimazolam and CNS7054, respectively. The results showed that remimazolam exhibited a high clearance of 15.9 (12.9, 18.2) mL/kg/min (median, Q25, Q75), a small central volume of distribution of 0.11 (0.08, 0.14) L/kg, and a short terminal half-life of 67 (49, 85) min. The context-sensitive half-time after a 4 h-infusion was 17 (12, 21) min. The metabolite CNS7054 showed a low clearance of 0.89 (0.33, 1.40) mL/kg/min, a small central volume of distribution of 0.011 (0.005, 0.016) L/kg, and a long terminal half-life of 321 (230, 770) min. In addition, elimination clearance of both remimazolam and CNS7054 increased with patient weight. When weight was standardized, pediatric pharmacokinetic characteristics were comparable to those observed in adults.

Dosing guidelines

Increasing single intravenous doses of remimazolam correlated with a proportional extension of mean sedation duration, ranging from approximately 8 minutes (at a dosage of 0.075 mg/kg) to around 40 minutes (at a dosage of 0.25 mg/kg) [39]. In healthy male volunteers who received a continuous intravenous infusion of remimazolam, the mean time to loss of consciousness was 5±1 minutes following the initiation of infusion, and the mean time to palinesthesia was 19±7 minutes after the cessation of infusion [41]. The incidence and duration of loss of consciousness were comparable between hepatic impairment subjects and healthy subjects; however, recovery was prolonged in patients with decreased liver function [42]. The current recommended dosage of remimazolam is 6-12mg /kg/h for the induction of general anesthesia. A remimazolam induction dose of 12 mg/kg/h led to a faster onset by about 15 to 20 seconds compared to a dose of 6 mg/kg/h. The recommended infusion rate of remimazolam for the maintenance of general anesthesia is 1-2 mg/kg/h. Remimazolam does not exhibit cumulative effects during general anesthesia. The timing of extubation was affected by the final infusion rate, the bispectral index score at the end of infusion, and sex, BMI, age, and plasma albumin concentration. However, it was not affected by the duration of surgery, total duration of anesthesia, and total dose of remimazolam, which may be attributed to its short context-sensitive half-time [43,44]. Notably, acute tolerance to remimazolam, as evidenced by changes in half-maximal inhibitory concentration (IC50), has been reported. Nevertheless, clinical data indicated that an infusion rate of 1 mg/kg/h was required to achieve adequate sedation, with no observed increase over time.

Due to its exceptionally low bioavailability and unique bitter taste, remimazolam is not appropriate for oral administration. Common administration routes in children include intranasal application, intravenous injection, and continuous intravenous infusion. Intranasal remimazolam exhibits onset of action within 5 min of administration and achieves peak drowsiness and relaxation within 10 to 20 min. However, the anxiolytic effect of intranasal remimazolam begins to weaken after 20 min of administration [45]. In this context, a study revealed that the ED95 of intranasal remimazolam for alleviating preoperative anxiety was 1.57 mg/kg for early childhood children and 1.09 mg/kg for preschool-aged children [21]. In contrast, when remimazolam is administered intravenously, the onset and peak duration are relatively shorter, requiring a reduced dosage. The 50% effective dose (ED50) and 95% effective dose (ED95) of intravenous remimazolam for alleviating preoperative anxiety were 0.051 mg/kg and 0.077 mg/kg, respectively, for pediatric patients aged 1-6 years [22]. Shen et al. [46] determined that the ED50 to achieve loss of consciousness after a single intravenous injection was 0.19 mg/kg in pediatric patients and recommended an anesthetic induction dose of 0.34 mg/kg for remimazolam during general anesthesia. A large cohort study involving 418 children (4.6±4.52 years) undergoing surgery showed that after intravenous infusion at a rate of 12 mg/kg/h for anesthetic induction and 1-2 mg/kg/h for anesthetic maintenance (mean duration: 109.4±83.1 min), the mean time from remimazolam discontinuation to awakening was 34.3±15.8 min [25]. Notably, the dosage regimen of remimazolam in children for procedural sedation and induction and maintenance of general anesthesia was based on the recommended dose for adults. Further research is needed to establish standardized dosing guidelines for remimazolam in children, particularly in special populations such as neonates and critically ill children.

Antagonist of remimazolam

Flumazenil, a benzodiazepine receptor antagonist, has been shown to be safe and effective in reversing midazolam-induced sedation, with a recommended dose of 0.01 mg/kg (maximum 1.0 mg) in pediatric patients [47]. However, it is not routinely used and is not even recommended for reversing the sedative effects of remimazolam. A major concern is the potential for re-sedation after a flumazenil bolus, particularly in patients who have received an overdose of remimazolam or suspected low total clearance of the drug [48]. In this respect, a report documented the case of a 62-year-old female undergoing laminoplasty for lumbar spinal stenosis, who received remimazolam at 1 mg/kg/h for 3.5 hours and experienced re-sedation 45 minutes after flumazenil reversal [49]. A meta-analysis performed by Wu et al. [50], which included nine RCTs and enrolled 745 participants from three nations, assessed the safety of remimazolam combined with flumazenil for post-general anesthesia recovery. The analysis revealed that the remimazolam-flumazenil combination resulted in faster recovery times, earlier extubation, reduced post-anesthesia care unit (PACU) duration, and a decreased likelihood of respiratory depression compared to propofol. Nevertheless, the rate of re-sedation after using remimazolam and flumazenil together was notably higher than that observed with propofol alone. These findings highlight the risk of re-sedation when using flumazenil to reverse remimazolam-induced sedation. The underlying mechanism is that the antagonistic effect of flumazenil depends on the concentrations of both flumazenil and benzodiazepines around the benzodiazepine receptors. Accordingly, close monitoring of postoperative consciousness and respiratory status is crucial in patients who receive remimazolam-flumazenil during general anesthesia. For patients with re-sedation, assess the depth of sedation and respiratory status while continuously monitoring vital signs. If profound respiratory depression occurs, endotracheal intubation may be required.

Procedural sedation outside the operating room

Procedural sedation is defined as a drug-induced, controlled state of sedation that reduces anxiety, pain, or discomfort while preserving spontaneous ventilation and protective reflexes, thus enabling the performance of diagnostic or therapeutic procedures. The number of examinations and surgeries performed under sedation in pediatric patients outside the operating room has surged significantly in the past 20 years. The required depth of sedation (including conscious sedation, deep sedation, and general anesthesia) for patients differs depending on the specific types of diagnostic and therapeutic procedures being performed. However, patients may progress from one level of sedation to another in response to changes in stimulus intensity during examinations and surgical procedures, which lead to inadequate or deeper sedation and increase the incidence of adverse events. Statistically, most adverse events during procedural sedation are airway and respiratory-related, such as airway obstruction, laryngospasm, respiration inhibition, and even apnea [51,52]. A comprehensive preoperative assessment, adequate preparation, trained staff, and close monitoring can significantly reduce the incidence of these adverse events. However, the selection of appropriate sedatives is equally important to ensure the safety of pediatric patients.

Chloral hydrate has long been the most used sedative in children for diagnostic procedures or surgeries [53-56]. However, it has a narrow therapeutic index and brings risks of respiratory depression and delayed sedation. Accordingly, the use of chloral hydrate has been heavily restricted in some countries, and in the United States, its manufacture has been halted [52,57]. Pentobarbital has a rapid onset but unpredictable duration of sedation, accounting for its low utilization rate in pediatric patients [52,58]. Midazolam is primarily employed for anxiolysis or in combination with fentanyl and ketamine to achieve moderate-to-deep sedation and analgesia. However, respiratory depression and agitation during recovery remain the main side effects [59-63]. Dexmedetomidine was once the most favored agent on account of its excellent sedation profile with minimal respiratory depression [64]. It is not widely used due to longer half-life and adverse effects of bradycardia and hypotension. Propofol remains the commonly used agent for procedural sedation despite its potential to suppress respiratory and circulatory functions. In recent years, remimazolam has been increasingly adopted for procedural sedation in children.

Imaging

Children, particularly infants and preschool-aged children, often require sedation to facilitate the completion of X/CT/MRI examinations or ultrasound examinations. There are few studies on the sedative effects of remimazolam in pediatric patients undergoing imaging examinations. A study evaluating the safety and efficacy of remimazolam during CT/MRI examinations in 120 children (mean age 2.8±1.3 years) showed that compared with the propofol group (initial dose 2 mg/kg, maintenance dose 2-3 mg/kg/h), the remimazolam group (initial dose 0.2 mg/kg, maintenance dose 1-2 mg/kg/h) exhibited a slightly longer onset time (50.81±0.23 s vs 42.11±0.19 s) but a shorter recovery time (8.21±2.52 min vs. 10.31±2.62 min) and more stable respiratory and circulatory parameters. The incidence of adverse events, including intraoperative body movement, dizziness, nausea, and vomiting, was comparable between the two groups [13].

For infantile echocardiographic examination, remimazolam also provides a better sedation profile. Yang et al. [14] recruited 46 infant patients aged 5-18 months requiring echocardiography to randomly receive remimazolam (0.3 mg/kg, iv) or dexmedetomidine (2 μg/kg, nasal drip) for sedation. The results demonstrated that the time to fall asleep and awakening time were shorter in infant patients administered remimazolam. Furthermore, there were no significant differences in vital signs or adverse effects between the two groups.

The use of remimazolam in pediatric imaging procedures offers several advantages. Its rapid onset and short recovery time make it an ideal choice for short-duration procedures, reducing the overall time spent in the imaging examination room and improving patient throughput. Besides, its favorable safety profile, particularly in terms of respiratory and cardiovascular stability, makes it a valuable option for children with comorbidities.

Endoscopy

The safety and efficacy of remimazolam for pediatric gastrointestinal endoscopy have also been studied. In a study involving 60 pediatric patients aged one to six years undergoing painless gastroscopy, remimazolam resulted in faster recovery times and a lower incidence of hypotension and injection pain compared to propofol [15]. Consistent with previous studies [13,65], the onset time of remimazolam (initial dosage of 0.2 mg/kg, iv) was longer than that of propofol (initial dosage of 2 mg/kg, iv). Notably, the remedial sedation rate and number of additional medications in the remimazolam group were significantly higher than that of the propofol group, which might be associated with the relatively low initial dosage of remimazolam administered. Another study showed that in pediatric painless gastroscopy, there was no difference between 0.3 mg/kg remimazolam and 3 mg/kg propofol in the sedation success, recovery time, and the incidence of body movements, bradycardia, and choking cough. However, remimazolam remained superior to propofol in terms of maintaining stable respiratory function [16]. Another study by Liu et al. [17] explored the application of different doses of remimazolam in children undergoing painless gastroscopy. A total of 165 children, aged 3 to 12 years, were randomly divided into four groups receiving intravenous injections of 2 mg/kg propofol, 0.2 mg/kg remimazolam, 0.3 mg/kg remimazolam, and 0.4 mg/kg remimazolam, respectively. The results indicated that 0.4 mg/kg remimazolam not only enhanced the success rate of sedation but also decreased the need for remedial sedation and the occurrence of adverse reactions. Furthermore, it did not prolong resuscitation time or increase the incidence of side effects. Overall, the above-mentioned effects could be achieved through a combination of remimazolam and propofol. Generally, combining medications can decrease the required drug dosage, lessen side effects, and leverage their unique strengths [66]. A recent study suggested that remimazolam combined with propofol for pediatric gastrointestinal endoscopy yielded a better sedative effect, less influence on hemodynamics, and fewer adverse reactions than using propofol only [18]. Accordingly, appropriate dosage optimization of remimazolam or the use of combined medication is crucial for improving the satisfaction levels of anesthetists, endoscopists, and patients.

Fiberoptic Bronchoscopy

Procedural Sedation for fiberoptic bronchoscopy presents a significant challenge, demanding adequate deep sedation to ensure a smooth examination and reduce the incidence of adverse events. In most cases, the safest, most controlled, and most frequently used approach of anesthesia in pediatric fiberoptic bronchoscopy is general anesthesia without tracheal intubation but with preserved spontaneous breathing and supplemented by topical anesthesia [67]. Previous studies have substantiated the safety and efficacy of remimazolam for procedural sedation in adults and aged patients during flexible bronchoscopy, with anesthesiologists reporting high satisfaction due to its quick onset and recovery, and minimal circulatory and respiratory depression [68-71]. In a trial of 50 children with a mean age of 5 years, Zhao et al. [72] evaluated the clinical efficacy of remimazolam combined with a laryngeal mask for pediatric patients requiring bronchial lavage. The study revealed no significant differences in sedative effect, recovery time, and incidence rate of adverse reactions between the remimazolam group (induction dose 0.2mg/kg, maintenance dose 1mg/kg/h) and the propofol group (induction dose 2mg/kg, maintenance dose 4-5 mg/kg/h). Chen et al. [19] compared remimazolam to propofol and remimazolam combined with low-dose propofol in 90 children undergoing fiberoptic bronchoscopy under deep sedation. Based on the intraoperative anesthetic drug regimen, the children were divided into three groups: group R (remimazolam 0.2-0.4 mg/kg), group P (propofol 1-3 mg/kg), and group RP (remimazolam 0.2 mg/kg, propofol 0.5 mg/kg). Consistent with their established profiles, group P showed the lowest blood pressure, heart rate, and SpO2 during the examination and exhibited the shortest induction and recovery times. Group R reported the highest cough scores. Remimazolam combined with low-dose propofol effectively balanced the strengths and weaknesses of remimazolam and propofol, ensuring more stable hemodynamics, a lower incidence of adverse reactions, and optimal surgical conditions in pediatric fiberoptic bronchoscopy.

Root Canal Therapy

Procedural sedation is essential for alleviating anxiety and panic and improving the cooperation of pediatric patients during outpatient dental treatment. Though numerous studies in pediatric dentistry investigated the safety and effectiveness of commonly used sedatives such as chloral hydrate, midazolam, ketamine, propofol, and so on, each drug or combination possesses distinct advantages and disadvantages [73,74]. Therefore, the search for a safe and effective sedation regimen in pediatric dentistry remains ongoing. The unique pharmacological profile of remimazolam, characterized by its rapid onset, short duration of action, and favorable safety margin, makes it particularly suitable for pediatric dentistry. In a clinical investigation conducted by Zhang et al. [20], the effect of remimazolam combined with propofol was evaluated for procedural sedation in pediatric patients undergoing outpatient root canal therapy. This study demonstrated that the combination of remimazolam (induction dose 0.2 mg/kg; maintenance dose 0.3 mg/kg/h) and propofol (induction dose 1-3 mg/kg; maintenance dose 6-12 mg/kg/h) compared to propofol alone (induction dose 1-3 mg/kg; maintenance dose 6-12 mg/kg/h) resulted in significantly shorter emergence times and reduced PACU stay duration, while effectively lowering the incidence of respiratory depression and decreasing total propofol consumption, without increasing the incidence of hypotension, bradycardia, body movement, or emergence agitation. Consistent with previous findings [19], this combination therapy prolonged sedation onset time. However, the safety and efficacy of remimazolam used alone for pediatric dental sedation have been largely understudied. In addition, frequent complications after discharge mainly include excessive somnolence, drug-specific motor imbalance, nausea, and emesis in pediatric dental sedation [75,76]. Whether remimazolam offers superior reduction of these complications compared to other sedatives warrants further investigation.

Premedication

Preoperative anxiety occurs in 50-75% of pediatric surgical patients and is clinically associated with postoperative behavioral changes [77]. Addressing preoperative anxiety in children is crucial for ensuring a smooth induction of anesthesia and minimizing the risk of postoperative behavioral issues [78]. Esketamine, midazolam, and dexmedetomidine have been demonstrated to be both safe and effective in alleviating preoperative anxiety and mitigating postoperative negative behaviors in pediatric patients [79-82], but they may prolong recovery and discharge times [83-85]. Dexmedetomidine reportedly has a longer onset of action and can induce deeper sedation [86]. In contrast, remimazolam (intranasal) demonstrates rapid onset (5.00± 1.47 min in the early childhood group, 4.68±1.33 min in the pre-school children group), as well as minimal hemodynamic and respiratory effects, and effectively alleviates preoperative anxiety through mild sedation in pediatric patients without delaying recovery and discharge [21,85]. Interestingly, Long et al. [21] observed distinct response patterns to remimazolam between early childhood and preschool-aged pediatric populations. Young children primarily exhibited smiling (42.50%) and quietness (82.50%), while preschool children showed smiling (32.50%), talkativeness (35.00%), quietness (47.50%), and sleeping (35.00%). The researchers attributed this phenomenon to variations in neurological development across different pediatric age groups. However, remimazolam results in poor compliance during intranasal administration due to significant nasal irritation [85]. Ni et al. [23] administered remimazolam via a mucosal atomizer device for inhalation combined with intranasal topical lidocaine pretreatment in preschool children to achieve preoperative sedation. This eliminated nasal burning and pain while reducing the dosage (i.e., the ED50 and ED95 of intranasal remimazolam for premedication were 0.65 and 0.78 mg/kg, respectively). For pediatric patients with established venous access, intravenous administration of remimazolam offers both convenience and satisfactory sedation efficacy. Wu et al. [24] enrolled 61 children aged 1-4 years scheduled for elective bilateral tonsillectomy with or without adenoidectomy under general anesthesia. The participants were randomly allocated into two groups to receive preoperative sedation with intravenous administration of 0.2 mg/kg remimazolam or 0.5 mg/kg esketamine. The study demonstrated that compared with esketamine, intravenous remimazolam for preoperative sedation exhibited a slightly prolonged onset time, significantly reduced recovery duration, and superior hemodynamic stability. Both groups exhibited comparable incidence rates of emergence agitation, postoperative adverse effects, and negative postoperative behavioral changes. In summary, the impact of remimazolam on alleviating preoperative anxiety has been established, although its effect on negative postoperative behavioral changes remains inconclusive due to the small sample size in current studies.

General anesthesia

Remimazolam distinguishes itself among various anesthetics due to its remarkable capacity to maintain stable hemodynamics during anesthesia, particularly in vulnerable patient populations, including the elderly, critically ill, and hypertensive individuals [87-92]. Meanwhile, it may reduce postoperative complications such as nausea/vomiting (PONV) and cognitive dysfunction in adults [93-95]. Given children’s heightened susceptibility to anesthesia-induced cardiovascular instability and their distinct pharmacokinetics, researchers have evaluated remimazolam’s safety in pediatric settings and whether children could also benefit from the administration of remimazolam during general anesthesia.

Safety and Adverse Effects

Kimoto et al. [25] conducted a study involving a large cohort of 418 pediatric patients, which represents the first analysis of remimazolam's clinical outcomes in pediatric anesthesia. During anesthesia, adverse events were observed in pediatric patients, with 75.2% (n=310/418) of patients experiencing mean arterial pressure deviations >20% from baseline, while only 5.0% (n=21/418) required ephedrine rescue, suggesting that most hemodynamic fluctuations were either transient or within clinically tolerable thresholds. However, a wide range of hemodynamic variability observed during general anesthesia cannot be unequivocally attributed to remimazolam's circulatory effects, given that other anesthetic agents (e.g., propofol, sevoflurane, ketamine) used during induction may also influence cardiovascular parameters. Another adverse event was PONV, with an incidence rate of 13%, lower than the overall incidence in pediatric patients [96]. In summary, the association between remimazolam and adverse events has not been elucidated, given the presence of confounding factors in this study.

The positive contribution of remimazolam in pediatric general anesthesia has been evidenced in later studies. A recent study showed that the efficacy and safety of remimazolam used for general anesthesia in preschool-age children was non-inferior to that of propofol, while fewer adverse events were documented [26]. Several studies from China have suggested that during general anesthesia in minor pediatric surgeries, remimazolam was associated with a lower incidence of hypotension and emergence delirium (ED) and a shorter awakening time than propofol [27-29]. Beyond its primary use for induction and maintenance of general anesthesia, remimazolam may act as an adjunct. The study found that both continuous infusion and single-dose injection of remimazolam during anesthesia could significantly reduce the incidence of ED in children following sevoflurane anesthesia. The continuous infusion of remimazolam can achieve an approximately 20% reduction in sevoflurane consumption, while a single bolus of remimazolam is more convenient and does not delay postoperative recovery compared with continuous infusion [30]. However, recent meta-analytic data suggests that evidence regarding its effects on ED and postoperative recovery remains inconclusive. For instance, Nitta et al. [31] reported that remimazolam might reduce ED incidence, but low evidence certainty and small sample sizes preclude definitive conclusions on delayed emergence. These inconsistent findings suggest that remimazolam's role in preventing or treating postoperative ED in pediatric patients, as well as its effect on recovery time, remains controversial. Therefore, further large-scale RCTs are required to determine the impact of remimazolam on ED and recovery time.

Applications in Specific Pediatric Populations

Remimazolam also exhibits a good safety profile for induction and maintenance of general anesthesia in specific pediatric patients. Pediatric patients with congenital cardiac disease are prone to circulatory collapse during general anesthesia resulting from the preexisting abnormal cardiac anatomy, limited compensatory mechanisms, and the cardiovascular depressant effect of anesthetics [97]. Remimazolam's established ability to minimize hypotension during general anesthesia suggests potential benefits for children undergoing congenital cardiac procedures. In a study by Huang et al. [32], 60 cardiac surgical children were randomized into remimazolam and propofol groups. The results demonstrated not only shortened ventilation and extubation times, with remimazolam combined with flumazenil, but also decreased hospital duration and expenses. Moreover, the mean arterial pressure and heart rate in the remimazolam group showed a lesser degree of decline compared to the propofol group after 3 minutes of administration. No difference in the incidence of adverse events was observed.

Remimazolam has been reported to be safe for administration in other high-risk pediatric patients. Malignant hyperthermia (MH) manifests as a life-threatening pharmacogenetic disorder triggered by depolarizing neuromuscular blockers (e.g., succinylcholine) or halogenated inhalational anesthetics (e.g., sevoflurane), characterized by uncontrolled skeletal muscle calcium release due to ryanodine receptor type 1 (RYR1) gene mutations, culminating in catastrophic hypermetabolism with characteristic triad: hypercapnia (EtCO₂>55 mmHg), metabolic acidosis (pH<7.2), and hyperthermia (core temperature >40°C). In this patient population, intravenous anesthetic agents such as propofol, ketamine, dexmedetomidine, or opioids and benzodiazepines are regarded as safe alternatives during anesthesia, thus choosing the appropriate anesthetics may reduce the incidence of MH. Petkus [33] reported a successful case in which remimazolam was used as an adjunct to propofol during total intravenous anesthesia (TIVA) for a six-year-old child with a family history suggestive of MH. No intraoperative complications were observed, highlighting the potential safety and efficacy of remimazolam in this high-risk population.

A report has indicated the safety of remimazolam for anesthetic induction and maintenance in a pediatric patient diagnosed with mitochondrial encephalomyopathy, lactic acidosis, and stroke-like episodes (MELAS). MELAS, as a rare mitochondrial disease, presents high anesthesia-associated risks, including MH related to inhaled anesthetics or depolarizing muscle relaxants, propofol infusion syndrome, hyperlactatemia, and increased sensitivity to non-depolarizing muscle relaxants. No epileptic seizures, delayed recovery, or other adverse effects occurred when remimazolam was used during general anesthesia in this 10-year-old girl with MELAS syndrome who underwent open gastrostomy. Interestingly, while occasional epileptic seizures were observed on the fifth postoperative day, the seizure frequency showed a significant reduction compared to the preoperative period [34].

Duchenne muscular dystrophy (DMD) is an X-linked recessive genetic disorder and a rare inherited neuromuscular disease, which predominantly occurs in male children and manifests as progressive and symmetrical muscle weakness. Patients with DMD exhibit increased sensitivity to sedatives, anesthetics, and neuromuscular blocking agents, which may lead to intraoperative and early postoperative cardiovascular and respiratory complications, as well as prolonged recovery time following anesthesia. These factors contribute to significant anesthetic risks in pediatric patients undergoing general anesthesia [98,99]. Horikoshi et al. [35] found that remimazolam could be used safely for the induction and maintenance of general anesthesia in a pediatric patient with DMD. Consistent with the pharmacokinetics of remimazolam, this child regained consciousness 20 minutes after drug discontinuation.

Glutaric acidemia type I (GA-1) is an autosomal recessive genetic disorder caused by a deficiency of glutaryl-CoA dehydrogenase (GCDH), leading to the accumulation of glutaric acid in cells. This condition can result in metabolic disorders and brain damage, with high rates of mortality and disability. However, only a few case reports have been published on the anesthetic and perioperative management of patients with GA-1. The key to managing GA-1 patients during anesthesia is avoiding hypercatabolism, which helps prevent encephalopathic crises. In pediatric patients with GA-1, inhalation anesthetics have been proven safe and effective, while propofol is not recommended due to the risk of infusion syndrome and severe metabolic acidosis [100, 101]. Currently, remimazolam is considered a novel alternative for general anesthesia in pediatric patients diagnosed with GA-1. In this regard, Tsuruno et al. [36] reported a case involving a four-year-old girl with GA-1 who underwent general anesthesia with remimazolam for a laparoscopic gastrostomy. The child exhibited stable hemodynamics throughout the procedure and exhibited no episodes of neuromuscular dysfunction or metabolic decompensation during the perioperative period. This case highlights the potential of remimazolam as a safe and effective anesthetic option for patients with GA-1, particularly in procedures requiring rapid recovery and minimal metabolic disruption.

Medium-chain acyl-CoA dehydrogenase deficiency (MCADD) is an autosomal recessive genetic disorder caused by a defect in mitochondrial medium-chain fatty acid beta-oxidation, leading to impaired energy metabolism. This condition primarily affects the heart, liver, skeletal muscle, and brain. Clinically, it can present with hypoglycemia, epilepsy, coma, and even death, particularly during periods of fasting or metabolic stress [102]. In pediatric patients with MCADD, the management of anesthesia requires careful consideration to avoid metabolic decompensation. Remimazolam has shown promise as a safe anesthetic option for stable MCADD pediatric patients, although its role in decompensated MCADD remains unclear [37].

Remimazolam demonstrates significant potential in reducing perioperative risks for high-risk pediatric patients through its favorable pharmacological profile, including excellent hemodynamic stability, minimal respiratory depression, and predictable ultra-short duration of action, making it particularly valuable for cardiac-compromised children and those with respiratory comorbidities. Furthermore, preliminary clinical evidence from case reports indicates that remimazolam exhibits a favorable safety profile in pediatric patients with genetic disorder-associated rare diseases, offering clinicians a novel option for anesthesia management in this special population. Despite limited evidence supporting remimazolam's use in pediatric general anesthesia, the increasing number of ongoing studies demonstrates its considerable potential for pediatric sedation and anesthesia (Table 3).

**Table 3:** Ongoing clinical trials of remimazolam in pediatric patients

Registration number	Title of the clinical trial	Ages eligible for study	Nation	Study start date	Status
NCT04851717	Investigation of Remimazolam in Children Undergoing Sedation for Medical Procedures	Aged 0-17	United States	15/11/2021	Unknown
NCT05527314	Effect of Remimazolam vs Sevoflurane Anesthesia on Incidence of Emergence Agitation and Complications in Children Undergoing Ophthalmic Surgery	Aged 3-8	China	23/08/2022	Completed
NCT06053489	Effect of Remimazolam and Sevoflurane Anesthesia on Recovery in Pediatric Patients	Aged 3-18	Korea	31/07/2023	Completed
NCT05990673	Dose of Remimazolam in Children	Aged 3-12	China	10/08/2023	Not yet recruiting
NCT05975255	Dose Finding Study for Remimazolam in Children	Aged 2-8	Korea	16/11/2023	Recruiting
NCT06170918	Dose of Remimazolam in Children for Intubation	Aged 3-12	China	20/12/2023	Not yet recruiting
NCT06121609	ED90 of Remimazolam Anesthesia Induction in Painless Bidirectional Endoscopy in Children	Aged 0-12	China	15/01/2024	Completed
NCT06214117	Comparison of Emergence Delirium: Remimazolam vs Sevoflurane Anesthesia	Aged 3-6	China	02/2024	Not yet recruiting
NCT06303037	Effect of Esketamine on 95% Induction Dose of Remimazolam	Aged 3-12	China	01/04/2024	Completed
NCT06698705	The Electroencephalogram Study Under Remimazolam Anesthesia in Children	Aged 1-12	China	22/11/2024	Recruiting
NCT06816173	Effect of Propofol Versus Remimazolam Intravenous Anesthesia on Respiratory Depression	Aged 3-6	China	02/2025	Not yet recruiting

Limitations and future directions

While these encouraging results demonstrate considerable clinical potential for remimazolam as a general anesthetic in pediatric populations, several limitations should be noted (Table 4). Currently, there are no large-scale multicenter RCTs investigating the safety, efficacy, and Pop PK/PD (population pharmacokinetics/pharmacodynamics) of remimazolam in children undergoing general anesthesia. Besides, the optimal dosage regimens for both induction and maintenance of general anesthesia in pediatric patients remain to be established, especially in the presence and interaction of opioids and muscle relaxants. In addition, current research on remimazolam has primarily focused on its application in gastrointestinal endoscopy, fiberoptic bronchoscopy, and minor surgical procedures, where its rapid onset and short duration of action are particularly advantageous. However, the metabolic profile and pharmacokinetic properties of remimazolam during prolonged continuous infusion remain poorly understood, especially in terms of potential metabolite accumulation and their clinical implications. Overall, the drug's potential application in longer surgical procedures and intensive care settings is worth considering. Furthermore, there are limited data available regarding the optimal dosing strategies for extended use, the potential development of tolerance, and the long-term safety profile in various patient populations. Additional studies are also needed to investigate the potential interactions between remimazolam and other concurrently administered medications during prolonged infusion, as well as its effects on different organ systems over extended periods.

**Table 4:** The application, medication methods, advantages, and limitations of remimazolam in pediatric populations FDA, Federal Drug Administration

Application	Medication methods	Advantages	Limitations
Imaging examinations	Intranasal route	Short onset of action, fast recovery, and no injection pain	Acute tolerance
Gastrointestinal endoscopy	Aerosol inhalation	Independence from hepatic and renal metabolism	Risk of re-sedation following flumazenil reversal of remimazolam
Fiberoptic bronchoscopy	Intravenous injection	Available specific antagonist	Unestablished long-term effects of remimazolam in children
Outpatient dental treatment	Continuous intravenous pumping	Minimal respiratory and cardiovascular depression	Lack of population pharmacodynamic study
Premedication	-	Reduces postoperative nausea and vomiting	Absence of evidence-based dosing guidelines for pediatric populations
Induction of general anesthesia	-	Reduces emergence delirium	Lacks FDA approval for pediatric populations
Maintenance of general anesthesia	-	No malignant hyperthermia risk observed	-

The side effects of anesthetic drugs remain a critical concern during their administration, as they can significantly impact patient safety and postoperative outcomes. In recent years, the neurotoxicity of anesthetics has become a research hotspot, especially in vulnerable populations such as pediatric patients and the elderly. Studies have shown that prolonged or high-dose exposure to certain anesthetics may lead to neuronal apoptosis, cognitive dysfunction, and long-term neurological deficits [103-105]. Therefore, the potential neurotoxicity of remimazolam and its associated short-term or long-term neurofunctional effects in pediatric patients require further research.

Case reports and limited studies have reported the safe use and advantages of remimazolam in specific pediatric populations. Large-scale clinical trials are required to establish standardized protocols for defined pediatric subgroups (e.g., neonates or critically ill children). However, critically ill children, neonates, and obese children differ significantly in physiology, pharmacokinetics, and anesthetic management compared to general pediatric patients. Conducting RCTs in these populations requires a careful risk-benefit assessment. Therefore, until the safety and efficacy of remimazolam are established in general pediatric patients, its use or clinical research in these high-risk pediatric populations should be approached with caution.

## Conclusions

Based on remimazolam’s pharmacological properties and current evidence, pediatric patients may benefit from its use. However, existing research in adults remains limited in scope and depth, and pediatric studies are even more limited. This limited evidence raises concerns about its widespread use in pediatric patients, especially in high-risk groups such as neonates, critically ill children, or those with decompensated metabolic diseases. Therefore, to ensure the safety of pediatric patients, the indications for remimazolam use in children should be rigorously defined and strictly controlled. Clinicians should carefully weigh the potential benefits against the risks, particularly when alternative anesthetic agents with more established safety profiles are available. Furthermore, further clinical trials are urgently required to evaluate remimazolam’s safety and efficacy in pediatric populations.

## References

[1] Rabbitts JA, Groenewald CB, Moriarty JP, Flick R (2010). Epidemiology of ambulatory anesthesia for children in the United States: 2006 and 1996. Anesth Analg.

[2] Tzong KY, Han S, Roh A, Ing C (2012). Epidemiology of pediatric surgical admissions in US children: data from the HCUP kids inpatient database. J Neurosurg Anesthesiol.

[3] Basel A, Bajic D (2018). Preoperative evaluation of the pediatric patient. Anesthesiol Clin.

[4] Lee C, Mason L (2006). Complications in paediatric anaesthesia. Curr Opin Anaesthesiol.

[5] Walkden GJ, Pickering AE, Gill H (2019). Assessing long-term neurodevelopmental outcome following general anesthesia in early childhood: challenges and opportunities. Anesth Analg.

[6] Chidambaran V, Costandi A, D'Mello A (2015). Propofol: a review of its role in pediatric anesthesia and sedation. CNS Drugs.

[7] Lanigan C, Sury M, Bingham R, Howard R, Mackersie A (1992). Neurological sequelae in children after prolonged propofol infusion. Anaesthesia.

[8] Bendiksen A, Larsen LM (1998). Convulsions, ataxia and hallucinations following propofol. Acta Anaesthesiol Scand.

[9] Trotter C, Serpell MG (1992). Neurological sequelae in children after prolonged propofol infusion. Anaesthesia.

[10] Costi D, Cyna AM, Ahmed S (2014). Effects of sevoflurane versus other general anaesthesia on emergence agitation in children. Cochrane Database Syst Rev.

[11] Zhang H, Li H, Zhao S, Bao F (2024). Remimazolam in general anesthesia: a comprehensive review of its applications and clinical efficacy. Drug Des Devel Ther.

[12] Gao YQ, Ihmsen H, Hu ZY (2023). Pharmacokinetics of remimazolam after intravenous infusion in anaesthetised children. Br J Anaesth.

[13] Fu H (2024). Comparison of the sedative effect of remazolam and propofol in the imaging examination of children. J Shandong First Med Univ Shandong Acad Med Sci.

[14] Haikou Yang JS, Qiao Wang (2021). Application comparison between remimazolam and dexmedetomidine sedation in infantile echocardiographic examination. Chongqing Med.

[15] Shi CC, Sun J, Zhang YF (2021). Application of remimazolam in children during painless gastroscopy. J Clin Pathol Res.

[16] Liu G, Qiao WN, Zhang JX (2023). Effects of remimazolam combined with alfentanil used for comfort gastroscopy in children. Chin J New Drugs Clin Rem.

[17] Liu HF, Wang JL, He YY (2024). The application of different doses of remazolam combined with alfentanil in painless gastroscopy in children. Prog Mod Biomed.

[18] Wu QX, Li Y, Meng W (2024). Efficacy of remimazolam combined with propofol for gastroscopy in children. J Clin Anesthesiol.

[19] Chen WJ, Bao WJ, Shi J (2024). Investigation into the application of remimazolamin conjunction with low-dose propofol for pediatric fiberoptic bronchoscopy. Sci Rep.

[20] Zhang TT, Xing F, Li Y (2022). Efficacy of remimazolam combined with propofol for sedation in pediatric patients undergoing outpatient dental root canal treatment. Chin J Anesthesiol.

[21] Long X, Wen LX, Yang H (2023). ED95 of remimazolam in nasal administration for attenuating preoperative anxiety in children. Front Med (Lausanne).

[22] Wu MC, Yang FF, Dai CX (2025). Determination of median effective dose of remimazolam for preoperative sedation in pediatric patients. Med J Chin PLA.

[23] Ni MJ, Jin YT, Wu QL (2024). Effective dose of intranasal remimazolam for preoperative sedation in preschool children: a dose-finding study using Dixon’s up-and-down method. Front Pharmacol.

[24] Wu MC, Yang FF, Ma XJ (2023). Comparison of clinical effects and safety of remidazolam and esketamine for preoperative sedation in children. J South Med Univ.

[25] Kimoto Y, Hirano T, Kuratani N (2023). Remimazolam as an adjunct to general anesthesia in children: adverse events and outcomes in a large cohort of 418 cases. J Clin Med.

[26] Fang YB, Zhong JW, Szmuk P (2025). Safety and efficacy of remimazolam tosilate for general anaesthesia in paediatric patients undergoing elective surgery: a multicentre, randomised, single-blind, controlled trial. Anaesthesia.

[27] Yu Y, Sun Q, Mao M (2023). Effect of remazolam and propofol on the anaesthetic effects of supernumerary tooth extraction in oral day surgery on children. Stomatology.

[28] Ｗu ZY, Zhao X, Zhang X (2023). Application effect of remimazolam on general anesthesia in children with dental caries during oral daytime surgery. Guangxi Med J.

[29] Wang J, Zhang YF, Yang WY (2024). Comparison of the anesthetic effects of remimazolam versus propofol in pediatric adenotonsillectomy. Chin J Rural Med Pharm.

[30] Cai YH, Zhong JW, Ma HY (2024). Effect of remimazolam on emergence delirium in children undergoing laparoscopic surgery: a double-blinded randomized trial. Anesthesiology.

[31] Nitta Y, Kamimura Y, Shiroshita A (2025). Concomitant use of intravenous remimazolam with Inhalation anesthesia and subsequent emergence delirium in children: a systematic review and meta-analysis. Cureus.

[32] Huang R, Zeng H, Li XL (2023). Effects of remimazolam combined with flumazenil on fast-track cardiac surgery in children. Chin J New Drugs Clin Rem.

[33] Petkus H, Willer BL, Tobias JD (2022). Remimazolam in a pediatric patient with a suspected family history of malignant hyperthermia. J Med Cases.

[34] Yamadori Y, Yamagami Y, Matsumoto Y (2022). General anesthesia with remimazolam for a pediatric patient with MELAS and recurrent epilepsy: a case report. JA Clin Rep.

[35] Horikoshi Y, Kuratani N, Tateno K (2021). Anesthetic management with remimazolam for a pediatric patient with Duchenne muscular dystrophy. Medicine.

[36] Tsuruno T, Tateiwa H, Hashimoto Y (2024). Remimazolam anesthesia for a pediatric patient with glutaric aciduria type I: a case report. Cureus.

[37] Kiyokawa M, Saito J, Nakai K (2022). A remimazolam and remifentanil anesthetic for a pediatric patient with a medium-chain acyl-CoA dehydrogenase deficiency: a case report. A A Pract.

[38] Dessai S, Ninave S, Bele A (2024). The rise of remimazolam: a review of pharmacology, clinical efficacy, and safety profiles. Cureus.

[39] Antonik LJ, Goldwater DR, Kilpatrick GJ (2012). A placebo- and midazolam-controlled phase I single ascending-dose study evaluating the safety, pharmacokinetics, and pharmacodynamics of remimazolam. Anesth Analg.

[40] Kempenaers S, Hansen TG, Van de Velde M (2023). Remimazolam and serious adverse events: a scoping review. Eur J Anaesthesiol.

[41] Schüttler J, Eisenried A, Lerch M (2020). Pharmacokinetics and pharmacodynamics of remimazolam (CNS 7056) after continuous infusion in healthy male volunteers. Anesthesiology.

[42] Stöhr T, Colin PJ, Ossig J (2021). Pharmacokinetic properties of remimazolam in subjects with hepatic or renal impairment. Br J Anaesth.

[43] Lohmer LL, Schippers F, Petersen KU (2020). Time-to-event modeling for remimazolam for the indication of induction and maintenance of general anesthesia. J Clin Pharmacol.

[44] Farag E, Shimamoto Y, Sanuki M (2022). Factors affecting prolonged time to extubation in patients given remimazolam. Plos One.

[45] Pesic M, Schippers F, Saunders R (2020). Pharmacokinetics and pharmacodynamics of intranasal remimazolam-a randomized controlled clinical trial. Eur J Clin Pharmacol.

[46] Shen Y, Sun Y, Wang YT, Peng ZZ, Bai J, Zheng JJ, Zhang MZ (2024). Dose equivalence of remimazolam and propofol for loss of consciousness in pediatric patients: a randomized clinical trial. Pain Physician.

[47] Peters JM, Tolia V, Simpson P (1999). Flumazenil in children after esophagogastroduodenoscopy. Am J Gastroenterol.

[48] Masui K (2022). Caution!! Reappearance of remimazolam effect after a flumazenil bolus: a larger bolus of flumazenil and a lower total remimazolam clearance are higher risks. J Anesth.

[49] Yamamoto T, Kurabe M, Kamiya Y (2021). Re-sleeping after reversal of remimazolam by flumazenil. J Anesth.

[50] Wu QT, Xu FC, Wang J (2023). Comparison of remimazolam-flumazenil versus propofol for recovery from general anesthesia: a systematic review and meta-analysis. J Clin Med.

[51] Ramaiah R, Bhananker S (2014). Pediatric procedural sedation and analgesia outside the operating room: anticipating, avoiding and managing complications. Expert Rev Neurother.

[52] Kamat PP, McCracken CE, Simon HK (2020). Trends in outpatient procedural sedation: 2007-2018. Pediatrics.

[53] Fong CY, Lim WK, Li L (2021). Chloral hydrate as a sedating agent for neurodiagnostic procedures in children. Cochrane Database Syst Rev.

[54] Delgado J, Toro R, Rascovsky S (2014). Chloral hydrate in pediatric magnetic resonance imaging: evaluation of a 10-year sedation experience administered by radiologists. Pediatr Radiol.

[55] Abdulhamid I, Tremblay M, Stenger J (2016). Chloral hydrate for sedation of children with asthma during dental treatment. Eur J Paediatr Dent.

[56] D'Agostino J, Terndrup TE (2000). Chloral hydrate versus midazolam for sedation of children for neuroimaging: a randomized clinical trial. Pediatr Emerg Care.

[57] Mason KP (2014). Challenges in paediatric procedural sedation: political, economic, and clinical aspects. Br J Anaesth.

[58] Teshome G, Belani K, Braun JL (2014). Comparison of dexmedetomidine with pentobarbital for pediatric MRI sedation. Hosp Pediatr.

[59] Roback MG, Carlson DW, Babl FE (2016). Update on pharmacological management of procedural sedation for children. Curr Opin Anaesthesiol.

[60] Plum AW, Harris TM (2015). Intranasal midazolam for anxiolysis in closed reduction of nasal fractures in children.. Int J Pediatr Otorhinolaryngol.

[61] Ma L, Jing Q, Wan K (2012). Evaluation of oral midazolam sedation for reducing dental fear in children with dental fear. West Chin J Stomatol.

[62] Monsereenusorn C, Malaithong W, Lertvivatpong N (2022). The efficacy and safety of midazolam with fentanyl versus midazolam with ketamine for bedside invasive procedural sedation in pediatric oncology patients: a randomized, double-blinded, crossover trial. Pediatr Hematol Oncol.

[63] Parker RI, Mahan RA, Giugliano D (1997). Efficacy and safety of intravenous midazolam and ketamine as sedation for therapeutic and diagnostic procedures in children. Pediatrics.

[64] Najafi N, Veyckemans F, Van de Velde A (2016). Usability of dexmedetomidine for deep sedation in infants and small children with respiratory morbidities. Acta Anaesthesiol Scand.

[65] Zhu HC, Su ZX, Zhou HM (2023). Remimazolam dosing for gastroscopy: a randomized noninferiority trial. Anesthesiology.

[66] Wang C, Gao YZ, Li J (2023). Safety and effectiveness of the combination of remimazolam tosilate and propofol in gastroscopy: a multicenter, randomized controlled, single-blind clinical trial. Front Pharmacol.

[67] Benjamin B (2000). Anesthesia for pediatric airway endoscopy. Otolaryngol Clin North Am.

[68] Pastis NJ, Yarmus LB, Schippers F (2019). Safety and efficacy of remimazolam compared With placebo and midazolam for moderate sedation during bronchoscopy. Chest.

[69] Zhou YY, Yang ST, Duan KM (2022). Efficacy and safety of remimazolam besylate in bronchoscopy for adults: a multicenter, randomized, double-blind, positive-controlled clinical study. Front Pharmacol.

[70] Zhang L, Yu L, Xu L (2023). Effectiveness of remimazolam besylate combined with alfentanil for fiberoptic bronchoscopy with preserved spontaneous breathing: a prospective, randomized, controlled clinical trial. Eur Rev Med Pharmacol Sci.

[71] Shi Y, Zhang L, Diao Y (2024). The effect of toluene sulfonic acid remimazolam on postoperative delirium in elderly patients undergoing painless bronchoscopy. Sci Rep.

[72] Zhao ZQ, Yang YY, Chen L (2022). A study on the feasibility and safety of remimazolam in combination with laryngeal mask airway for pediatric fiberoptic bronchoscopy-assisted bronchoalveolar lavage. New Mom Newborn.

[73] Nathan JE (2022). Comparisons of varying dosages of chloral hydrate-hydroxyzine with and without meperidine for managing challenging pediatric dental behavior: a retrospective study of 35 years of sedation experiences. J Clin Pediatr Dent.

[74] Inchingolo F, Inchingolo AM, Ferrante L (2024). Pharmacological sedation in paediatric dentistry. Eur J Paediatr Dent.

[75] Huang A, Tanbonliong T (2015). Oral sedation postdischarge adverse events in pediatric dental patients. Anesth Prog.

[76] Dosani FZ, Flaitz CM, Whitmire HC (2014). Postdischarge events occurring after pediatric sedation for dentistry. Pediatr Dent.

[77] Kassai B, Rabilloud M, Dantony E (2016). Introduction of a paediatric anaesthesia comic information leaflet reduced preoperative anxiety in children. Br J Anaesth.

[78] Watson AT, Visram A (2003). Children's preoperative anxiety and postoperative behaviour. Paediatr Anaesth.

[79] McGraw T, Kendrick A (1998). Oral midazolam premedication and postoperative behaviour in children. Paediatr Anaesth.

[80] Chen S, Lang B, Wu L (2023). Efficacy and safety of oral versus intranasal midazolam as premedication in children: a systematic review and meta-analysis.. Minerva Anestesiol.

[81] Pant D, Sethi N, Sood J (2014). Comparison of sublingual midazolam and dexmedetomidine for premedication in children. Minerva Anestesiol.

[82] Peng K, Wu SR, Ji FH (2014). Premedication with dexmedetomidine in pediatric patients: a systematic review and meta-analysis. Clinics.

[83] Viitanen H, Annila P, Viitanen M (1999). Premedication with midazolam delays recovery after ambulatory sevoflurane anesthesia in children. Anesth Analg.

[84] Parnis SJ, Foate JA, van der Walt JH (1992). Oral midazolam is an effective premedication for children having day-stay anaesthesia. Anaesth Intensive Care.

[85] Cai YH, Wang CY, Fang YB (2024). Preoperative anxiolytic and sedative effects of intranasal remimazolam and dexmedetomidine: a randomized controlled clinical study in children undergoing general surgeries. Drug Des Devel Ther.

[86] AlSarheed MA (2016). Intranasal sedatives in pediatric dentistry. Saudi Med J.

[87] He MF, Gong CJ, Chen YN (2024). Effect of remimazolam vs. propofol on hemodynamics during general anesthesia induction in elderly patients: single-center, randomized controlled trial. J Biomed Res.

[88] Dai QC, Zhao JL, Miao XY (2024). Effects of different doses of remimazolam on hemodynamics during general anesthesia in patients with septic shock. Eur Rev Med Pharmacol Sci.

[89] Huang YQ, Yan T, Lu GT (2023). Efficacy and safety of remimazolam compared with propofol in hypertensive patients undergoing breast cancer surgery: a single-center, randomized, controlled study. BMC Anesthesiol.

[90] Katsuragawa T, Mimuro S, Sato T (2023). Effect of remimazolam versus sevoflurane on intraoperative hemodynamics in noncardiac surgery: a retrospective observational study using propensity score matching. JA Clin Rep.

[91] Tsuchiya M, Kuwabara K, Mizutani K (2025). Anaesthesia with combination of remimazolam and flumazenil has advantages over desflurane including faster recovery and fewer effects to inhibit haemodynamics during MitraClip procedure. Eur J Anaesthesiol.

[92] Muñoz-Carrillo JL, Rodríguez-Cortes N, Lévano ST (2024). Remimazolam versus propofol in general anesthesia of complex surgery in critical and non-critical patients: meta-analysis of randomized trials. J Clin Med.

[93] Lee SC, Jung JW, Choi SR (2023). Comparison of postoperative nausea and vomiting incidence between remimazolam and sevoflurane in tympanoplasty with mastoidectomy: a single-center, double-blind, randomized controlled trial. Medicina.

[94] Yoo YM, Park JH, Lee KH (2024). The incidences of nausea and vomiting after general anesthesia with remimazolam versus sevoflurane: a prospective randomized controlled trial. Korean J Anesthesiol.

[95] Kuang Q, Zhong N, Ye C (2023). Propofol versus remimazolam on cognitive function, hemodynamics, and oxygenation during one-lung ventilation in older patients undergoing pulmonary lobectomy: a randomized controlled trial. J Cardiothorac Vasc Anesth.

[96] Frelich M, Sklienka P, Romanová T (2024). The effect of BIS-guided anaesthesia on the incidence of postoperative nausea and vomiting in children: a prospective randomized double-blind study. BMC Anesthesiol.

[97] Friesen RH (2014). Anesthetic drugs in congenital heart disease. Semin Cardiothorac Vasc Anesth.

[98] Lian XH, Jing Y, Luo T (2025). Pharmacological interventions for the management of anesthesia and sedation in patients with Duchenne muscular dystrophy: a systematic review and meta-analysis. Front Med.

[99] Muenster T, Mueller C, Forst J (2012). Anaesthetic management in patients with Duchenne muscular dystrophy undergoing orthopaedic surgery. Eur J Anaesthesiol.

[100] Tsiotou AG, Malisiova A, Bouzelos N (2011). The child with glutaric aciduria type I: anesthetic and perioperative management. J Anesth.

[101] Teng WN, Lin SM, Niu DM (2014). Anesthetic management of comprehensive dental restoration in a child with glutaric aciduria type 1 using volatile sevoflurane.. J Chin Med Assoc.

[102] Allen C, Perkins R, Schwahn B (2016). A retrospective review of anesthesia and perioperative care in children with medium‐chain acyl‐CoA dehydrogenase deficiency. Paediatr Anaesth.

[103] Slikker WJ, Zou XJ, Hotchkiss CE (2007). Ketamine-induced neuronal cell death in the perinatal rhesus monkey. Toxicol Sci.

[104] Jevtovic-Todorovic V, Hartman RE, Izumi Y (2003). Early exposure to common anesthetic agents causes widespread neurodegeneration in the developing rat brain and persistent learning deficits. J Neurosci.

[105] Hogarth K, Tarazi D, Maynes JT (2023). The effects of general anesthetics on mitochondrial structure and function in the developing brain. Front Neurol.

